# Development of the DADSS* Breath Alcohol Sensor System for Automobiles: Technical Design and Human Participant Testing

**DOI:** 10.3390/s26092685

**Published:** 2026-04-26

**Authors:** Kianna Pirooz, Timothy Allen, Rebecca Spicer, Sam Kalmar, Jing Liu, Jane McNeil, Gordana Vitaliano, Scott E. Lukas

**Affiliations:** 1 KEA Technologies Inc., 1 Monarch Drive Suite 100, Littleton, MA 01640, USA; kianna.pirooz@keatechinc.com (K.P.); tim.allen@keatechinc.com (T.A.); 2 Impact Research, LLC., 7170 Riverwood Dr., Suite A, Columbia, MD 21046, USA; 3 Behavioral Psychopharmacology Research Laboratory (BPRL), McLean Imaging Center, McLean Hospital, Belmont, MA 02478, USAliujing@mclean.harvard.edu (J.L.); gvitaliano@mclean.harvard.edu (G.V.)

**Keywords:** alcohol, ethanol, automobile, drunk driving, impairment, BrAC, BAC, human

## Abstract

**Highlights:**

**Main findings**

- Using a novel contactless infrared sensor combined with CO_2_ measures, alcohol can be rapidly detected during passive breathing and the concentration correlates well with blood alcohol content.
- The sensor system is compact enough to fit in the steering console of an automobile.

**Implications of the main findings**

- During the testing many participants were unaware that they were over the legal limit for driving a motor vehicle.
- By interfacing this device with the electronics of a vehicle, the incidence of drunk driving may decrease.

**Abstract:**

Despite many efforts to curtail drunk driving, alcohol-related traffic fatalities and injuries continue to be a major public health problem in the United States (U.S.) and most of the world. Technologies exist that prevent an automobile from starting if the driver’s breath alcohol exceeds 20 milligrams per deciliter (mg/dL), but these devices are only fitted to vehicles of individuals who have been convicted of Driving Under the Influence (DUI). A new approach must be taken to reduce the incidence of drunk driving by integrating an alcohol sensor system in vehicles as part of the delivered hardware. The system must be fast, accurate, and contactless—meaning that a forced exhalation is not required to measure the concentration of alcohol on the breath. We report on a novel device, the Driver Alcohol Detection System for Safety (DADSS) Breath Alcohol Sensor System, which uses the mid-infrared region of the electromagnetic spectrum to concurrently monitor alcohol and expired carbon dioxide (CO_2_) to accurately quantify the breath alcohol concentration in samples that have been diluted in the atmosphere before being measured. The system was validated in a research laboratory with 70 male and female volunteers in 187 individual study days. Participants were given various doses of alcohol to consume and then breath and blood samples were collected simultaneously. Pearson correlation coefficients between the DADSS Breath Alcohol Sensor system and blood samples indicate a strong correlation between the measures, with an overall Pearson correlation of 0.8875 over an alcohol concentration range of 0–220 mg/dL. These results indicate that incorporating the DADSS system into motor vehicles has the potential to reduce the incidence of drunk driving.

## 1. Introduction

Excessive alcohol consumption is responsible for approximately 178,000 deaths and ~4 million years of potential life lost (YPLL) in the United States each year [1]. Binge drinking (consuming 4 or more drinks per occasion for women; 5 or more drinks per occasion for men) is responsible for more than half of the deaths and two-thirds of the YPLLs due to excessive drinking [2] and is associated with many health and social problems, including alcohol-impaired driving, interpersonal violence, risky sexual activity, and unintended pregnancy [3,4]. Most people under age 21 who drink, report binge drinking 2–3 times per week [5,6].

Impaired driving continues to be the leading contributor to roadway deaths in the United States. In 2023 alone, alcohol-related crashes resulted in 12,429 fatalities [7]. Emerging alcohol detection technologies are being developed to help counter this trend. In response, major global automakers have partnered through the Automotive Coalition for Traffic Safety (ACTS) with the U.S. federal government, specifically the National Highway Traffic Safety Administration (NHTSA), forming a public–private initiative known as the Driver Alcohol Detection System for Safety (DADSS). This collaborative effort is focused on creating an innovative in-vehicle safety system designed to significantly reduce and ultimately eliminate alcohol-impaired driving. Alcohol impairs areas of the brain that are most relevant to operating a motor vehicle and include slowed reaction/braking times, the ability to remain in the travel lane, and the inability to recognize potentially dangerous or risky situations that are developing in real-time [8,9,10]. Moreover, some individuals exhibit these disruptions at BACs below the legal limit of 80 mg/dL. Individuals who display less impulse control and have higher levels of sensation-seeking display more risky driving [11,12], which is exacerbated when individuals are intoxicated.

While the overall rate of driving under the influence has decreased since 2002 [13], the incidence of drunk driving has started to climb since 2019 and remains a serious public health problem [14]. In 2023, there were 12,429 fatalities on the U.S. highways [7] that involved drunk driving, which equates to 34 deaths every day, or one person every 42 min. Furthermore, alcohol-related crashes resulted in an estimated 497,000 injuries and $68.9 billion in economic costs in 2019, which makes up 20% of all crash costs [14]. Of those motor vehicle crashes, those with alcohol concentrations above 80 mg/dL or higher account for more than 90% of the economic costs and harm to society [14]. The economic costs encompass lost productivity, hospital and legal costs, rehabilitation services, and property damage. Many of the individuals who contribute to these losses are repeat offenders, resulting in either the death of themselves or another individual, serious injury to them, or damage and destruction to property. Although public attention often centers on fatalities caused by alcohol-impaired driving, the scope of harm extends far beyond deaths, with substantial injuries and widespread damage to both private and public property:

1. The proportion of roadway deaths involving alcohol varies significantly by state, from 21% in Utah to 40% in Texas [7].
2. In 2023, 25% of traffic deaths among children aged 14 and younger involved a driver who was impaired by alcohol [7].
3. The total annual economic burden associated with drunk driving in the United States is estimated at $340 billion [15].
4. According to the FBI’s Crime Data Explorer, in 2024, approximately 730,943 people were arrested for operating a motor vehicle while under the influence of alcohol [16].

Drugs other than alcohol (e.g., marijuana, cocaine) are involved in approximately 18% of motor vehicle driver deaths. These other drugs are often used in combination with alcohol [17], but there are no quick methods of determining the blood concentration of these illicit drugs.

A 2022 report from the Traffic Injury Research Foundation [18] noted that U.S. drivers who reported being “very or extremely concerned” about drunk driving fell from 65% in 2021 to 59% in 2022. They further noted that the number of drivers who drove when they thought they were over the legal BAC limit decreased from 22.5% in 2021 to 19.6% in 2022, and of these respondents, 9.3% thought they would not get caught, and 30.7% thought that despite being over the legal limit they were still okay to drive.

These findings are particularly significant because alcohol-related roadway deaths are widely believed to be underestimated [19], in part because many states do not mandate blood alcohol testing for every fatal crash. Both controlled laboratory experiments and real-world driving studies show that most drivers, including those with substantial experience, exhibit marked impairment at a blood alcohol concentration (BAC) of 80 mg/dL. This impairment affects essential driving functions such as braking, steering, changing lanes, decision-making, and managing divided attention [20,21] that contribute to significant increases in lane position and lane crossings [22,23]. At this concentration, performance typically declines by roughly 40% to 60% compared with a sober state. Evidence further indicates that one of the most critical deficits involves a reduced capacity to manage multiple tasks simultaneously [9,23,24], which is a fundamental requirement for safe vehicle operation.

Clearly, the efforts to curtail driving while under the influence have not been universally effective. We report on the development of the Driver Alcohol Detection System for Safety (DADSS) Breath Alcohol Sensor System, which aims to help prevent impaired driving by accurately and quickly detecting alcohol concentrations in drivers. Auto manufacturers and NHTSA will be responsible for implementing and regulating, respectively, the system and deciding how the automobile will be programmed to respond once alcohol is detected. Furthermore, they will determine the threshold of alcohol concentration that triggers an action. The DADSS system differs from traditional interlock systems in the following important ways: (1) the sensor is unobtrusive; (2) the DADSS system does not require that the driver provide a forced exhalation sample into a mouthpiece—the breath is sampled passively and continuously as soon as the driver enters the vehicle; (3) if the system detects a poor quality breath sample (such as if the driver is wearing a mask), the system may require a brief directed breath sample towards the sensor; (4) processing time is a matter of seconds, resulting in little to no delay in being able to operate the vehicle (assuming the BAC is below the limit).

**Figure 1:**
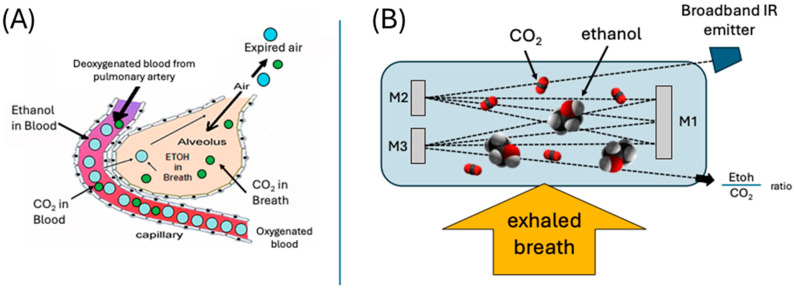
Fundamental principles of a breath-based alcohol detection. (**A**) Anatomical and physiological components of the exchange of gases between blood and air in the lungs. Gases and volatile compounds move from areas of higher to lower concentrations by diffusing across the membrane that separates capillaries from the alveolar sacs in the lungs. (**B**) Operational principles of the DADSS Breath Alcohol Sensor System to mimic the normal physiological exchange of ethanol and CO_2_. The beam of a broadband infrared emitter is reflected multiple times and detects the presence of both ethanol and CO_2_, resulting in an ethanol/CO_2_ ratio.

**Figure 2:**
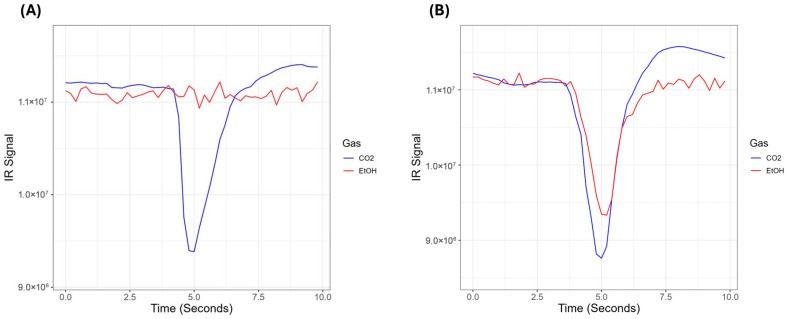
IR signal as a function of time (seconds) (**A**) in a sober participant, and (**B**) in a dosed participant.

The DADSS breath alcohol sensor system uses proprietary technology (covered under US patent 11,143,646, as well as others) to concurrently measure CO_2_ and ethanol (EtOH) concentrations in human breath. The sensor is a compact high-resolution nondispersive infrared (NDIR) sensor that uses two detectors, a CO_2_-specific detector and an EtOH-specific detector, that measure the absorbance of light in the mid-infrared range of the electromagnetic spectrum (2.5–25 μm) (Figure 1). The detectors measure two specific wavelengths of light that correspond to characteristic “fingerprint” absorption bands of the two gases. The characteristic absorbance used to detect EtOH has been chosen such that the measured absorbance is in a region where the absorbance of IR light by water does not interfere with the detection of EtOH. The sensor measures IR absorbance at a frequency of five hertz, allowing it to accurately measure the transient rise and fall of ethanol and CO_2_ concentrations due to a driver’s breath and to separate that signal from the background signal of the environment. Figure 2 shows the representative absorbances of a participant’s breath versus time (A) without and (B) with ethanol.

This design allows the system to analyze alcohol in breath within the vehicle cabin without the driver having to specifically provide a deep-lung breath sample or use a mouthpiece. The working principle of the sensor is to use measurements of expired CO_2_ as an indication of the degree of dilution of the alcohol in expired air. The normal concentration of CO_2_ in ambient air is low (~350–450 ppm), and the CO_2_ concentration in alveolar air is known, predictable, and remarkably constant. Humans absorb oxygen from the surrounding air and exhale CO_2_, and the amount that is exhaled is rather constant at about 5% (50,000 ppm) [25]. When CO_2_ is exhaled, it is quickly mixed with the surrounding air, but the ratio of ethanol to CO_2_ remains unchanged. By measuring both CO_2_ and alcohol at the same time, the system can account for dilution effects through a mathematical correction. As described by Hök (2006), the relationship between measured CO_2_ and alcohol concentrations, combined with the known concentration of CO_2_ in alveolar air, can be used to estimate the alcohol concentration in alveolar breath [26]. The addition of the CO_2_ monitoring helps ensure that accurate sampling of the expired air has occurred. One important benefit of using NDIR and this design is its long-term stability, which removes the need for repeated calibration over the lifespan of the system. The manufacturer specifies that the device has a 15-year lifetime, which is supported by their validation data.

Finally, the issue of partition coefficient was addressed in the system’s design. The partition coefficient is the ratio of the concentration of a compound (in this case, ethanol) in a mixture of two immiscible phases while at equilibrium. Thus, this ratio measures the difference in ethanol solubility in the two phases. The two phases are blood and air, as the “Gold Standard” for alcohol determination in the body is venous blood. All breath-based devices measure the ethanol concentration in air, and ethanol’s partition coefficient can vary according to anatomical, physiological, and environmental factors. It has been reported that the partition coefficient of ethanol in humans ranges between 1128:1 and 2989:1 [27]. The partition coefficient has been adopted in the United States to 2100 mg ethanol/dL blood per mg ethanol/dL air. Quantitative evidential breath alcohol analyzers are currently factory calibrated in grams of ethanol per 210 L of breath, thus equating to a partition coefficient ratio of 2100:1, which is what the DADSS Breath Alcohol Sensor System uses.

Initial benchtop testing was completed before the sensors were used in the human participant experiments. Sensors were tested at a range of temperatures at breath alcohol (BrAC) levels of 0, 20, and 80 mg/dL using simulated breaths in a chemistry lab setting, but the sensors were not calibrated or adjusted in any way before they were used, if they met the standards for proceeding with testing. The only excluded sensors had performance that suggested they had been damaged during transport to the lab.

Once benchtop testing was complete, the sensors were delivered to McLean Hospital for human testing. The goal of the human participant testing was to ensure that the sensors are accurate in a real-world situation. Blood alcohol content (BAC) is the accepted medium among established medical and legal guidelines, with 80 mg/dL (0.08%) being the accepted legal limit for operating a motor vehicle in most states in the United States. States have adopted lower limits for commercial drivers, while some have adopted zero tolerance for underage individuals. The DADSS Breath Alcohol Sensor System can be tuned to any threshold (e.g., between zero and 80 mg/dL).

## 2. Materials and Methods

### 2.1. DADSS Breath Alcohol Sensor System—Hardware

Table 1 depicts the breath sampling sensor and details each generation of the DADSS Breath Alcohol Sensor System (developed by Senseair AB (“Senseair”) Delsbo, Sweden). Human participant testing was conducted only with Generations 3.0–3.3; Generation 4.0 is expected to be released in late 2024.

### 2.2. DADSS Breath Alcohol Sensor System—Software

The Senseair Xpira^©^ software (versions 1.0–3.2.3) was designed to monitor and collect data from the DADSS Breath Alcohol Sensors during studies. Infrared (IR) signals are collected at 5 Hz, and BrAC results are calculated within moments of the breath sample being collected. If the software detected an unsuccessful breath sample, the participant was instructed to repeat the breath (exhalation). All breath data collected by the DADSS Breath Alcohol Sensor System were de-identified and only referenced by a unique study identifier.

### 2.3. Participants

A total of 70 healthy adult male and female volunteers between the ages of 21–55 were recruited via online advertisements to participate in the studies, for which they were compensated. Most individuals participated in more than one experiment, providing within-participant comparisons. The study protocol and consent procedures were reviewed and approved by the Mass General Brigham Institutional Review Board. Before enrollment, all individuals underwent comprehensive medical and psychiatric screening that included blood chemistry, liver function tests, EKG, urinalysis, and drug and alcohol screens; all female participants received a pregnancy test that had to be negative in order to continue in the study. Eligibility required that participants did not meet DSM-5 criteria for any psychiatric or substance use disorder. Additional exclusion criteria included uncontrolled or unstable medical conditions, significant central nervous system disorders such as multiple sclerosis or cerebrovascular events, as well as diagnoses including major depressive disorder, bipolar disorder, schizophrenia, psychotic disorders, or other organic mental conditions. Written informed consent was obtained from all participants. These rigorous standards were applied to ensure that it was safe for the volunteers to receive a test dose of ethyl alcohol and have up to 1/2 of a unit of blood withdrawn. On each test day, they received a breath alcohol test (Alco-Sensor FST; Intoximeter, Inc., St. Louis, MO, USA), a twelve-panel urine toxicology screen (CliaWaived Inc.^®^, San Diego, CA, USA or AmediCheck^®^, Hayward, CA, USA), and a urine pregnancy test if female (QuPID^®^ hCG Pregnancy Test, Hangzhou, Zhejiang, China)—all had to be negative before the study could proceed. Starting in 2020, eligible participants also had to provide a COVID-19 antigen test (FlowFlex^®^, San Diego, CA, USA), which also had to be negative before proceeding with study activities.

### 2.4. General Procedure

The overall procedure was conducted similar to prior alcohol administration studies [28]. Participants were instructed that they could not drink alcohol for 36 h prior to the scheduled study visit, smoke cigarettes after 10 pm the night before the study visit or eat food after 8 pm. On the study day, participants arrived at the laboratory (via taxicab, rideshare, or other public transportation) after an overnight fast (no food after 8 pm; only non-caffeinated fluids in the morning before arriving) and were provided with a standardized breakfast (juice and toast). This small amount of food is necessary to eliminate nausea and possible vomiting that occurs after consuming alcohol on an empty stomach. Participants were not permitted to drive themselves to the laboratory but were instead required to take a rideshare or taxicab.

**Figure 3:**
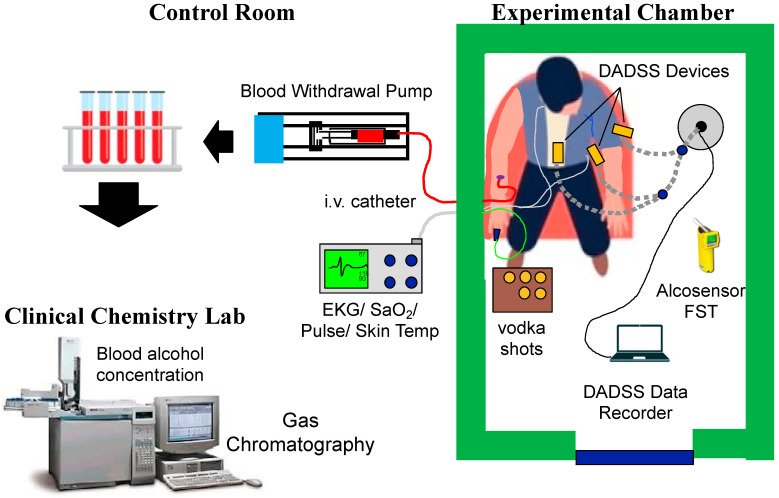
Layout of the Human Psychopharmacology Research Laboratory where human participant testing was conducted (arrows depict the movement of the blood samples from the participant to the clinical chemistry lab). The various DADSS Breath Alcohol Sensor Systems were mounted on a microphone stand to allow up to three sensors to be tested simultaneously. All DADSS sensors were controlled using a single laptop running the Senseair Xpira software. Individual’s vital signs were continuously monitored, and blood was sampled via an i.v. catheter outside of the test chamber. Once the study was over, the blood samples were transferred to the Clinical Chemistry Laboratory (one floor below the lab) for BAC quantification.

**Figure 4:**
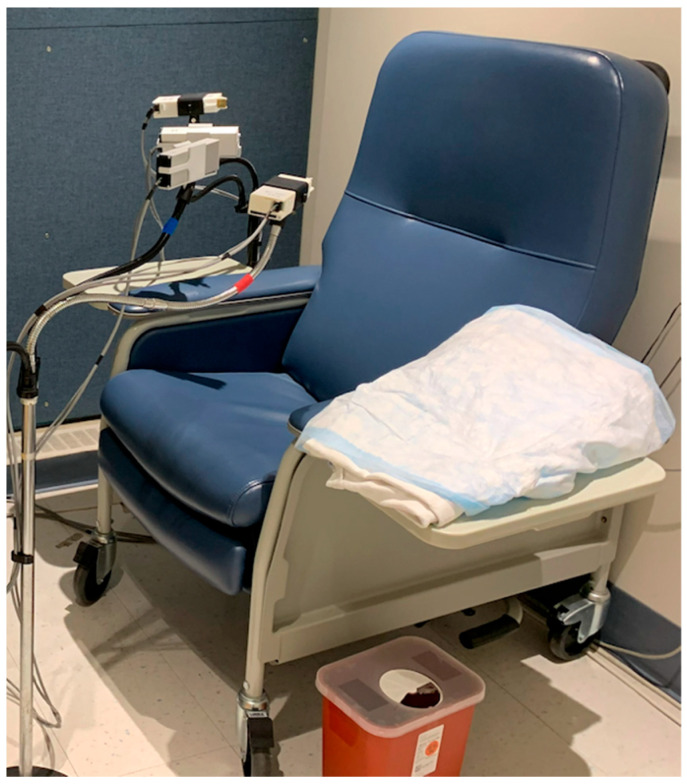
Photograph of the testing environment with the DADSS sensors oriented in several locations in front of the participant’s face.

On each study day, an indwelling intravenous catheter (Dakmed-Kowarski ThromboResistant Catheter, Buffalo, NY, USA) was placed in the participant’s arm and connected to an exfusion syringe pump set to withdraw blood continuously at a rate of 1 mL per minute. Participants remained seated in a recliner chair throughout the procedure while a blood sample was collected every 2 or 5 min and exhaled normally into the DADSS breath alcohol sensors (Figure 3 and Figure 4). A photo of the arrangement showing the placement of the various sensor units mounted on microphone stands (Figure 4). Comparable time locked breath samples were also collected via a reference breath sample (Alco-Sensor FST; Intoximeter). Participants’ vital signs (heart rate, oxygen saturation, skin temperature, respiration, and EKG) were recorded continuously using a patient monitor (Atlas Vital Signs Monitor, Welch-Allyn, Inc., Skaneateles Falls, NY, USA). The testing protocols over time involved simulating a variety of drinking scenarios such as while exercising, eating a snack, eating a full meal, combined with an energy drink, single bolus doses, multiple drinks simulating binge drinking, etc. The data from these scenarios will be presented in a different manuscript as they focus on the biology and drinking patterns of human volunteers. The focus of the present study is on the DADSS Breath Alcohol Sensor System and the parameters under which the hardware and software operate. As such, the drinking scenario type is irrelevant to the data or its interpretation.

At time zero, the participants drank the alcohol beverage (see below), and all measurements continued as before until the end of the study (on average, 3 h). Participants were given a set of questionnaires (Subjective High Assessment Scale (SHAS)) [29] to rate their subjective mood state and degree of intoxication approximately every 20 min. Sample questions included: “On a scale of zero to 10, with zero being ‘not at all’ and 10 being ‘extremely’, how drunk do you feel right now?” Additional questions included items like how “Good,” “Bad,” “Floating,” “Hungry,” “Nauseous,” etc. Two additional questions were added at the end that asked how safe they would feel driving a motor vehicle (on the 0–10 scale) and asked them if they would drive right now, “Yes” or “No”? After the study ended, the i.v. catheter and electrodes were removed, and the participant was escorted to a waiting area and given lunch and non-alcoholic beverages. They were assessed approximately every 20 min and were required to remain in the laboratory until their BrAC dropped to below 50 mg/dL and they were able to pass a field sobriety test.

### 2.5. Alcohol Dosing

Alcohol dosing was weight-based, ranging from 0.3 to 0.9 g/kg. Beverages containing vodka were freshly prepared prior to administration, and participants followed standardized instructions regarding both volume and pace of consumption, either as a single large dose or divided into three drinks over a 90-min period. For a small number of experiments, participants were dosed using light beer (~5% alcohol content) and wine (~12% alcohol content), but the doses were calculated to achieve the same target g/kg dose as with the vodka. When dosing using vodka, drinks were consumed as straight shots or mixed with orange or cranberry juice. Beer and wine drinks were consumed as drinks in 16-oz plastic cups. Because the focus of these studies was on comparing the BAC to the DADSS breath (BrAC) values, the rate and dose of alcohol administration for each participant were not relevant, as researchers were only interested in comparing the x-y pairs collected at the same time.

### 2.6. Blood Alcohol Content (BAC)

Blood alcohol concentrations were analyzed in the BPRL laboratory using a previously established method [30,31]. Blood samples were collected at intervals of two or five minutes and transferred into 6 mL gray top BD Vacutainer tubes containing sodium fluoride and potassium oxalate to preserve sample integrity. Tubes were inverted ten times to ensure adequate mixing. Whole-blood samples were processed using an internal standard solution of n-propyl alcohol prepared in sulfuric acid, resulting in a concentration of 160 mg per dL, and a 10% aqueous sodium tungstate solution was used to precipitate proteins [32]. Samples were then mixed using a vortex mixer for 20 to 30 s and centrifuged at 4650 rpm for 30 min at 25° Celsius. Following centrifugation, 100 µL of the clear supernatant was transferred into autosampler vials with crimp caps. A 0.5 µL aliquot was injected into a gas chromatograph, specifically the Agilent 7890A Gas Chromatograph (Agilent Technologies, Santa Clara, CA, USA), and analyzed using capillary chromatography with flame ionization detection [33,34]. The intra-assay coefficient of variation was 1.10%, and the inter-assay coefficient of variation was 1.96%.

### 2.7. DADSS Breath Alcohol Sensor System Measurements

At designated intervals of approximately two or five minutes, participants were also asked to exhale toward DADSS breath sensors, allowing for passive sample collection without direct contact. These measurements were timed to occur simultaneously as a blood sample was collected from the participant. Frequent pairings of referential breath samples from the Alco-Sensor FST were also scheduled. Four generations of the DADSS Breath Alcohol Sensor System were tested over seven years and were designated Gen 3.0, 3.1, 3.2, and 3.3. (Table 1). As a new generation of the sensor was available, testing with the previous generation was phased out, while testing with the new generation was phased in.

### 2.8. Reference Breath Samples

For comparison, breath alcohol measurements were simultaneously obtained using the Alco-Sensor FST, a handheld research-grade instrument. Instructions on providing a proper forced breath sample to the Alco-Sensor FST unit are given to participants by research staff trained in using the instrument. Briefly, participants are asked to take a deep breath and blow directly into the mouthpiece at a steady force for at least 8 s. Inadequate flow or too short duration results in an error and would require the sample to be repeated. On the morning of each study, a trained operator checked the unit’s accuracy using an alcohol dry gas standard made by Intoximeter^®^ per the instructions outlined in the manual. The device was calibrated if not within the acceptable error specifications (±0.002) and checked again for accuracy. A comprehensive log was kept with the date and time of accuracy checks and unit calibrations.

### 2.9. Data Analysis

Because of the large volume of data points collected from each sensor, the data was aggregated to adopt a more extensive statistical approach to accomplish the study’s aims. For each participant, each DADSS sensor BrAC measurement was paired with a BAC measurement taken within three minutes of the breath measure, where possible. Using the aggregated data, the mean, and standard deviation of the DADSS sensor BrAC and BAC measurements were compared. Univariate linear regression modeling examined the association of DADSS sensor BrAC with BAC among the pairs. The Pearson correlation coefficient was computed to examine the strength and direction of the correlation between BrAC and BAC.

## 3. Results

### 3.1. Correlation Between Breath- and Blood-Based Alcohol Concentrations

From November 2015 to August 2023, 187 individual study days were conducted with 70 participants. Participants were permitted to participate in multiple different study scenarios as long as the study days were conducted 48 h apart and they still met the study eligibility requirements. These participants ranged from ages 21 to 55, with a mean age of 29, and 75% under age 32. About two-thirds (64%) of participants were male. Overall, 9163 DADSS BrAC measurements were paired with a BAC measurement that was taken within three minutes of the breath measurement. Fifty-three percent of these pairs (n = 4851) were with generation 3.0 sensors, 15% (n = 1372) with generation 3.1, 25% (n = 2319) with generation 3.2, and 7% (n = 621) with generation 3.3.

**Figure 5:**
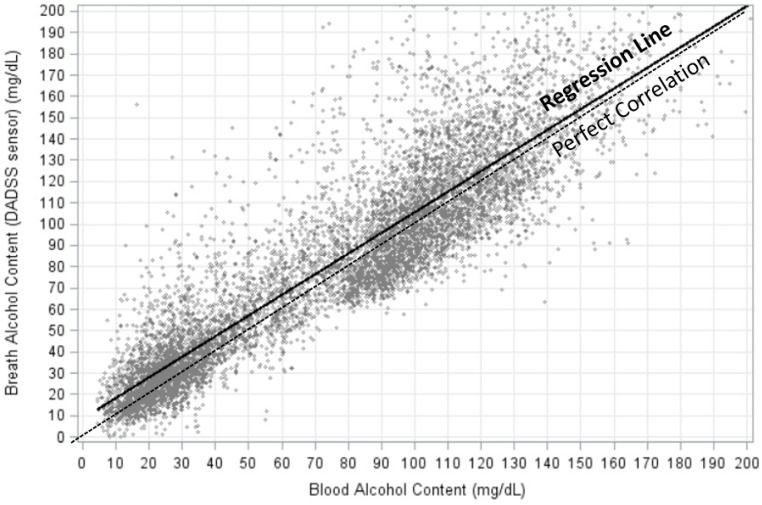
Linear association of alcohol concentrations as measured with the DADSS Breath Alcohol Sensor System versus BAC as measured via gas chromatography. Univariate linear regression (solid black line) of DADSS BrAC as a predictor of BAC revealed a significant (*p* < 0.0001) linear relationship between the two measures (predicted BAC = 9.0191 + 0.8097 * BrAC) and an r-square of 0.7877. n = 9090.

Initial analyses were performed to compare the alcohol concentrations from each of the four generations of the DADSS Breath Alcohol Sensor System. While small but significant differences among the sensor generations were found in terms of correlations with one another and with blood, the DADSS breath measures were pooled for this analysis. Univariate linear regression of DADSS BrAC as a predictor of BAC revealed a significant (*p* < 0.0001) linear relationship between the two measures (predicted BAC = 9.0191 + 0.8097 * BrAC) and an r-square of 0.7877. A scatterplot of the DADSS BrAC measures (n = 9090) paired with positive BAC measures (73 BAC = 0 pairs not included) is presented in Figure 5 and includes the regression line describing the relationship.

Pearson correlation coefficients between the DADSS Breath Alcohol Sensor System and BAC positive samples by generation indicate a strong positive correlation between the measures, with an overall Pearson correlation of 0.8875 (95%CI: 0.8831, 0.8918). These results demonstrate that the correlation between the DADSS Breath Alcohol Sensor System and the gold standard, blood alcohol, is extremely good, but they also identify a slight offset in the alignment between the two techniques. Therefore, we aligned the values to one another in order to adjust for the slight overestimation of BrAC by the DADSS Breath Alcohol Sensor System.

### 3.2. Time Course of Alcohol Concentration After Oral Administration

**Figure 6:**
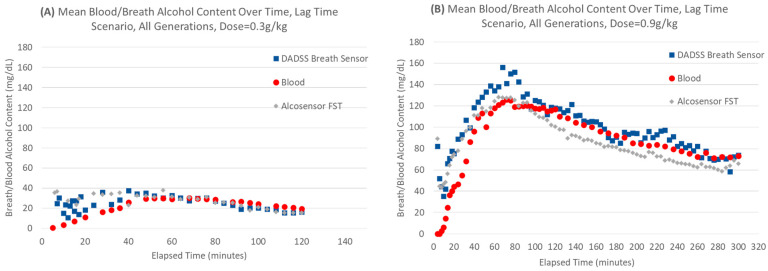
Time course of alcohol concentration in the DADSS breath sensor, blood, and the Alco-Sensor FST in volunteers after consuming (**A**) 0.3 or (**B**) 0.9 g/kg of ethyl alcohol. The DADSS breath data are aligned based on the ratio of DADSS BrAC mean versus blood BAC mean by generation.

Alcohol is rapidly absorbed from the small intestines, especially when drinking on an empty stomach. Figure 6 depicts the time course of alcohol concentration through the various phases of absorption, distribution, metabolism, and elimination in a cohort of male and female participants. The DADSS Breath Alcohol Sensor System BrAC measurements have been aligned based on the ratio of DADSS BrAC mean versus blood BAC by DADSS sensor generation to account for a systematic overestimation of BrAC when alcohol is present in the blood (BAC > 0). Figure 6 illustrates how residual alcohol from buccal (i.e., “mouth alcohol”) can be seen right after dosing, but this dissipates within 15–20 min after drinking. Once the buccal alcohol has disappeared, the time course of alcohol concentration depicted by the two breath-based systems matches the blood extremely well.

### 3.3. Subjective Reports of Intoxication

**Figure 7:**
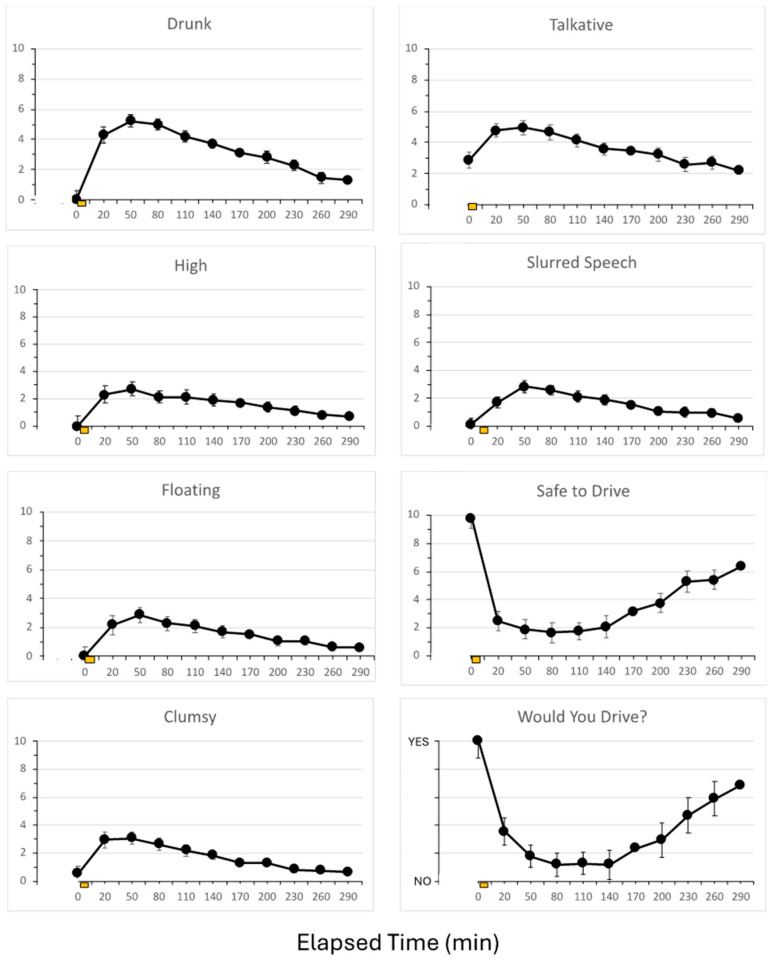
Subjective reports of mood state and confidence in the ability to drive a motor vehicle as a function of time. Values are mean ± standard error of the mean (sem) from 17 participants who consumed 0.9 g/kg of alcohol over 10 min, starting at time zero. Ordinate scale dimensions are 0 for “not at all” and 10 for “extremely”. For the question “Would you Drive?”, the scale was coded as 0 for the answer “No” and 1 for the answer “Yes”.

The participants were asked to complete a series of visual analog scales asking about their degree of intoxication, mood state, and confidence in their driving ability. Results for all questions followed the pattern that has been well-documented after acute alcohol consumption. A selected set of questions is depicted in Figure 7. showing that the effects paralleled the rise and fall of alcohol concentration, as depicted in Figure 6. Acute consumption of alcohol resulted in an expected rise in reports of how “Drunk,” “Talkative,” “High,” Slurred Speech,” “Floating,” and “Clumsy” that peaked around 50 min after dosing and then declined to baseline ratings. One of the more interesting questions related to the participant’s confidence in their driving and how that related to breath alcohol concentration. Nearly 30% of the participants indicated that they felt “Safe to Drive” a vehicle even though they were legally under the influence of alcohol at 80 mg/dL.

The other questions asked in the SHAS related to how “Nauseated, “Anxious,” “Dizzy,” Uncomfortable,” “Tired,” “Sleepy,” “Bored,” and “Confused” they felt did not change appreciably during the experiment.

## 4. Discussion

### 4.1. Importance of High Correlations Between Breath and Blood and Direct Applications of the DADSS Breath Alcohol Sensor System

Many prior studies have demonstrated a good correlation between breath and blood alcohol concentrations, but the breath samples have all been collected via a forced exhalation procedure designed to collect a “deep lung sample.” While the commercial products marketed as alcohol “sniffers” that are incorporated into flashlights for law enforcement exist (e.g., the PAS IV Flashlight Passive Alcohol Tester, AlcoPro, Morrisville, NC, USA), these devices are meant to be swept inside a vehicle to detect alcohol on a person’s breath [35]. However, these devices will also pick up alcohol in the atmosphere inside the vehicle, regardless of who was drinking, as well as the alcohol in the air that has escaped from an open container. In contrast, the DADSS Breath Alcohol Sensor System not only has excellent correlations with blood, but it does so in the absence of a mouthpiece and without a deep forced exhalation sample. In essence, it is a “contactless” system that targets the driver of the vehicle. The present approach is novel in that it relies on a well-established relationship between exhaled CO_2_ as a marker for dilution of passive breathing. This strategy sets this system apart from every other breath-based device in use today, as each exhalation is analyzed for both alcohol and CO_2_ simultaneously.

### 4.2. The Physiology of Breath Sampling and Its Relationship to Blood Concentrations and the Associated Engineering Challenges

One of the key aspects of using breath samples as a surrogate for blood alcohol concentrations is the partition coefficient of alcohol in air and water. However, for breath to accurately reflect the concentration in blood, the breath must be sampled from deep lung space, close to the alveolar sacs where the actual exchange takes place. In order to design a system that can function just as accurately from a passive breath sample, the DADSS program developed a system that capitalizes on the concentration of expired CO_2_ with every passive breath. The concentration of CO_2_ varies in the environment, but is quite stable in the human body, at approximately 5% [25].

### 4.3. Correlations Between BAC, BrAC, and Subjective Ratings of Intoxication

One key finding of the present study is that individuals still reported that they would operate a motor vehicle even though their breath alcohol concentration was 80 mg/dL or higher. This finding alone justifies the need for a system like DADSS because individuals are not very good at estimating their level of impairment [36]. In addition, individuals who have had a DUI report greater confidence in their ability to drive after drinking [37]. Contrary to popular belief among the lay public and the non-specialist professional, casual observations of an individual are not reliable indicators of the degree of intoxication. Many factors contribute to an individual’s outward appearance after consuming alcohol, and the effects are both dose- and time-dependent. Individuals with a high BAC can often perform relatively simple motor tasks, especially when they are in a familiar situation or are not subjected to challenging scenarios. This concept has two important implications: (1) an intoxicated person will report feeling far more sober than they really are, and (2) casual observers will misidentify these individuals as being less intoxicated than they really are. But when an individual is challenged and must perform even a simple task, or their balance is challenged by a small change in the surface, or finds themselves in a novel environment, then their ability to complete the task or maintain balance is significantly impaired, and their performance suffers [28,38].

### 4.4. DADSS Breath Alcohol Sensor System—In-Vehicle Applications for Research

**Figure 8:**
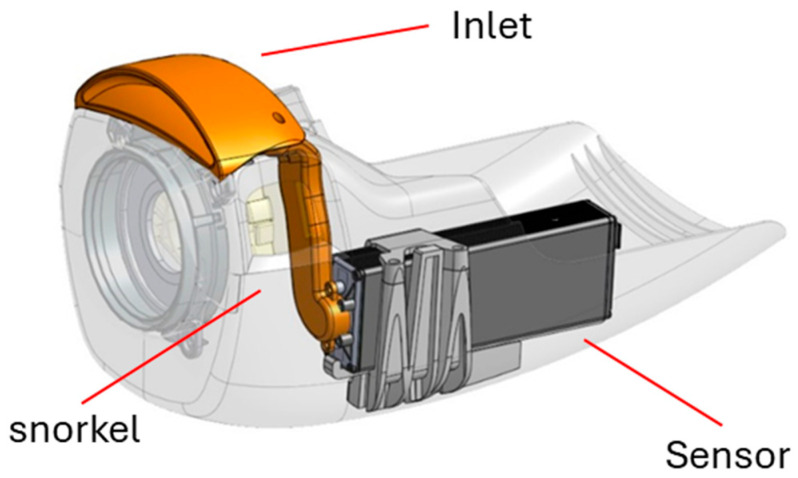
Alcohol and CO_2_ detection sensors for placement in motor vehicles.

There are several ways in which the sensor could be integrated into a vehicle for validation and verification activities. The individual unit could be adapted and customized to fit into any domestic and commercial vehicles. Figure 8 shows a design example of how the DADSS sensor system (sensor, snorkel, and inlet) has been integrated into the steering column of a vehicle.

**Figure 9:**
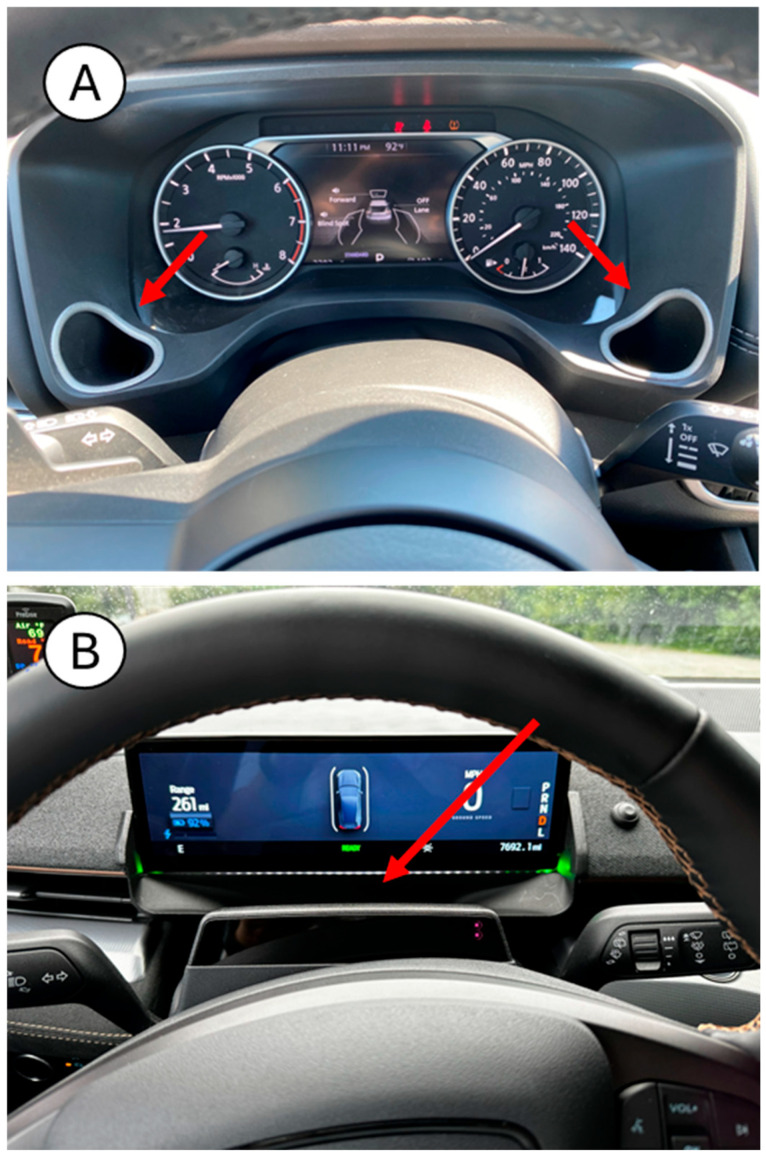
Alcohol- and CO_2_-detection sensors integrated in select automobile models (red arrows mark sensor location). (**A**) Instrument Panel sensor in Nissan Rogue and (**B**) Instrument Panel sensor in Ford Mach-E.

Figure 9 shows some of the research-based integrations that have been conducted thus far on various car models. Some of these integrations are accompanied by LEDs that provide the user with feedback on their breath result. The unobtrusive inlet for the breath sampling sensor is located on top of the steering column, as the red arrow indicates).

Because the system is designed to be adapted to all automobile makes and models, each vehicle manufacturer would determine the actual final configuration in the cabin.

## 5. Conclusions

We report on the performance and human testing of the Driver Alcohol Detection System for Safety (DADSS) Breath Alcohol Sensor System, which is a compact contactless sensor that quantifies breath alcohol concentrations in a matter of seconds and accurately predicts alcohol concentrations in blood. Validations were accomplished via laboratory-based human participant testing protocols, during which volunteers consumed various doses of beverage-grade alcohol and then their breath and blood were simultaneously tested for alcohol concentration. The correlation between breath and blood are exceptionally accurate over a broad range of alcohol concentrations from zero to 220 mg/dL. These data also demonstrate that individuals are not aware that they are over the legal limit of 80 mg/dL and often report that they would feel comfortable operating a motor vehicle. The DADSS Breath Alcohol Sensor System is a promising technology that will help reduce the incidence of drunk driving.

## 6. Patents

The technology reported here is covered by ACTS–owned patents and pending patents including:

Canada: 2,920,796; 2,925,806; 2,881,817; 2,881,814; 3,010,352; 2,987,729

China: ZL201280042179.6; ZL201480047728.8; ZL201480055848.2; ZL202010449254.7; ZL201680083149.8; ZL201680086043.3; ZL201680046009.3; ZL20128019106

Europe: 3038865; 2888587; 2888588; 3433611; 3304045; 2683569; 3994015

Japan: 6553614; 6656144; 6496244; 6408991; 6954875; 7138047; 6786624; 7028768; 6121916

South Africa: 2016/00797; 2016/01639; 2015/01247

Sweden: 536784; 536782; 544050; 543554; 544897; 545663

U.S: 10,099,554; 11,001,142; 10,710,455; 9,281,658; 11,391,724; 10,151,744; 11,143,646; 10,826,270; 11,104,227; 9,823,237; 11,874,262; 11,072,345; 11,513,070; 11,862,934; 8,479,864

## Figures and Tables

**Table 1:** Evolution of the various generations of the DADSS Breath Alcohol Sensor System with specifications and key design elements.

	Gen 1.0	Gen 2.0	Gen 3.0	Gen 3.1	Gen 3.2	Gen 3.3	Gen 4.0
Intended use	Research PoC	First field trial, indoor usage	First vehicle field PoC	After market	Vehicle PoC	After market	Automotive
Design focus	Multipass optical cell	Robustness & resolution	Size reduction	Resolution	Resolution	Robustness	Robustness & resolution
Operation mode	Directed exhalation	Directed exhalation	Directed exhalation	Directed exhalation	Directed exhalation	Directed exhalation	Directed exhalation. Passive monitoring
Resolution (µg/L)	7.5	1.2	1.5	1.0	0.7	0.7	0.3
Exhalation distance	<2 cm	<5 cm	<5 cm	<10 cm	<40 cm	<40 cm	<60 cm

## Data Availability

The original contributions presented in this study are included in the article. Further inquiries can be directed to the corresponding author(s).

## Abbreviations

The following abbreviations are used in this manuscript:

BAC	Blood Alcohol Content
BrAC	Breath Alcohol Content
CO_2_	Carbon Dioxide
DADSS	Driver Alcohol Detection System for Safety
mg/dL	milligrams per deciliter
ppm	parts per million
